# Response Rate and Completeness of Questionnaires: A Randomized Study of Internet Versus Paper-and-Pencil Versions

**DOI:** 10.2196/jmir.9.3.e25

**Published:** 2007-09-30

**Authors:** Sissel Marie Kongsved, Maja Basnov, Kurt Holm-Christensen, Niels Henrik Hjollund

**Affiliations:** ^3^Department of Clinical Social MedicineInstitute of Public HealthAarhus UniversityAarhusDenmark; ^2^Department of RadiologyRanders Regional HospitalRandersDenmark; ^1^Department of Clinical Social MedicineCentre of Public HealthCentral Denmark RegionAarhusDenmark

**Keywords:** Questionnaire design, random allocation, Internet, postal service, evaluation, data collection methodology

## Abstract

**Background:**

Research in quality of life traditionally relies on paper-and-pencil questionnaires. Easy access to the Internet has inspired a number of studies that use the Internet to collect questionnaire data. However, Internet-based data collection may differ from traditional methods with respect to response rate and data quality as well as the validity and reliability of the involved scales.

**Objective:**

We used a randomized design to compare a paper-and-pencil questionnaire with an Internet version of the same questionnaire with respect to differences in response rate and completeness of data.

**Methods:**

Women referred for mammography at a Danish public hospital from September 2004 to April 2005, aged less than 67 years and without a history of breast cancer, were eligible for the study. The women received the invitation to participate along with the usual letter from the Department of Radiology. A total of 533 women were invited to participate. They were randomized to receive either a paper questionnaire, with a prepaid return envelope, or a guideline on how to fill in the Internet-based version online. The questionnaire consisted of 17 pages with a total of 119 items, including the Short Form-36, Multidimensional Fatigue Inventory-20, Hospital Anxiety and Depression Scale, and questions regarding social status, education level, occupation, and access to the Internet. Nonrespondents received a postal reminder giving them the option of filling out the other version of the questionnaire.

**Results:**

The response rate before the reminder was 17.9% for the Internet group compared to 73.2% for the paper-and-pencil group (risk difference 55.3%, *P* < .001). After the reminder, when the participant could chose between versions of the questionnaire, the total response rate for the Internet and paper-and-pencil group was 64.2% and 76.5%, respectively (risk difference 12.2%, *P* = .002). For the Internet version, 97.8% filled in a complete questionnaire without missing data, while 63.4% filled in a complete questionnaire for the paper-and-pencil version (risk difference 34.5%, *P* < .001).

**Conclusions:**

The Internet version of the questionnaire was superior with respect to completeness of data, but the response rate in this population of unselected patients was low. The general population has yet to become more familiar with the Internet before an online survey can be the first choice of researchers, although it is worthwhile considering within selected populations of patients as it saves resources and provides more complete answers. An Internet version may be combined with the traditional version of a questionnaire, and in follow-up studies of patients it may be more feasible to offer Internet versions.

## Introduction

Research in quality of life traditionally relies on paper-and-pencil questionnaires. Internet surveys may have advantages compared to the traditional paper-and-pencil surveys with respect to turn-a-round time, expenses, and data management [1]. However, Internet-based data collection may differ from traditional methods with respect to response rate and data quality as well as validity and reliability of the involved scales. Only a few studies have systematically evaluated Internet-based survey methods. The main questions have addressed validity [2-7], response rate, response speed, and completeness of data [1,6-14].

Most studies report small differences in answers obtained in Internet and paper-and-pencil versions of questionnaires [2-7]. Pealer et al found no significant difference in response rates, the Internet version having a response rate of 62% compared to 58% for the paper-and-pencil version [12]. Ritter et al found a high response rate in both groups of a study population recruited on the Internet: 87% in the Internet group, and 83% in the paper-and-pencil group [9]. These studies either recruited their participants on the Internet or invited only participants with a known active email account, and, as a consequence, the results from these studies are not valid for a general population of patients. A Swedish study conducted in a general population sample obtained a response rate of 50% in the Internet group and 64% in the paper-and-pencil group. The method included two reminders, including a contact by telephone [6]. However, in a workplace health survey, a poor response rate was observed among the Internet group (19%) compared to the paper-and-pencil group (72%), but this study did not include a reminder procedure [1].

Overall, the results with respect to differences in response rate are inconsistent, which may reflect differences in methodology and populations. We have not identified any randomized studies comparing Internet and paper-and-pencil questionnaires in patient populations unselected with respect to Internet access. Therefore, we aimed to evaluate an Internet survey method in comparison to paper-and-pencil with respect to response rate and completeness of data in a randomized controlled design among women referred for mammography.

## Methods

Participants were women referred for mammography from September 2004 to April 2005 in the Department of Radiology at the public hospital, Randers Regional Hospital. The municipality of Randers has around 62000 inhabitants. Patients were referred by their family doctor. A consultant at the Department of Radiology assigned the referred patients to one of three categories: acute, subacute, or nonacute. Subsequently, a letter was sent to the woman informing her about the date, location, and other details of the mammography. The women were randomized to be invited to answer either an Internet version or a paper-and-pencil version of a questionnaire. We only invited women up to retirement age (67 years in Demark) who did not have a history of breast cancer. Patients from all categories (acute, subacute, or nonacute) were invited until February 2005, whereafter only patients in the acute and subacute groups were invited to participate.

**Figure 1:**
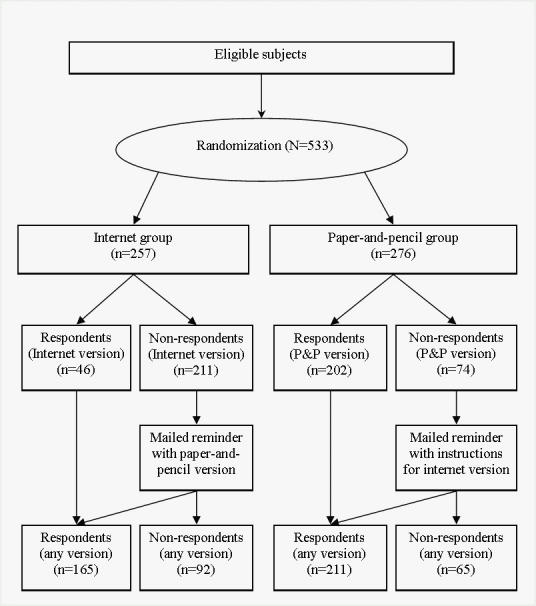
Flow of participants through the randomized trial

Nonrespondents in both groups were mailed a reminder after 10 days, given that the date of their mammography was not reached. The reminder informed the woman that she was free to answer the opposite version of the originally requested questionnaire if she so desired. Only questionnaires filled in before the date of the mammography were included in the analysis. The procedure is outlined in Figure 1. There were no incentives to promote the survey response.

**Figure 2:**
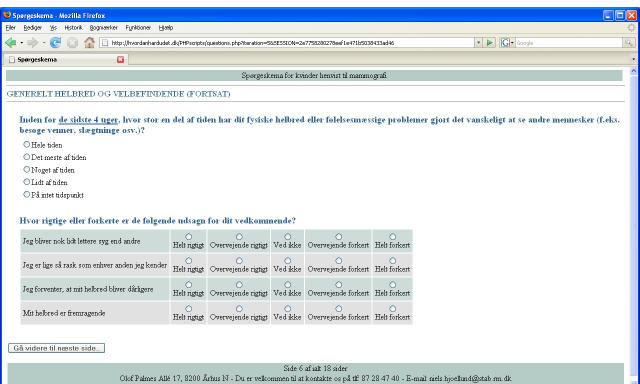
Screenshot of the Internet version of the questionnaire

**Figure 3:**
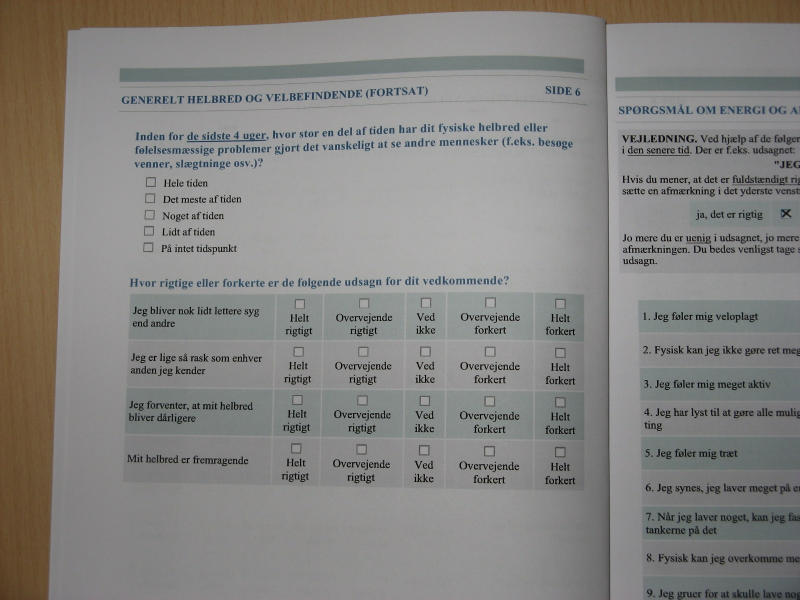
Photograph of the paper-and-pencil version of the questionnaire

The letter to women randomized to answer the paper-and-pencil version included a paper questionnaire and a prepaid return envelope, while the letter to women randomized to answer the Internet version included a guideline on how answer the Web-based version. Access to the Internet questionnaire required entry of a unique five-letter username. No password was needed since the first letter in the username was a redundancy code. The layout of the Internet version was as close to the paper version as possible (see Figure 2 and Figure 3). In the Internet version, the participants were reminded of missing answers if they tried to leave a page incomplete. However, after pressing an “OK” button, they were allowed to continue even if there were still missing answers [15]. The questionnaire consisted of 17 pages and 119 items and included Short Form-36 [16], Multidimensional Fatigue Inventory-20 [17], and The Hospital Anxiety and Depression Scale [18]. Questions regarding social status, education level, occupation, and access to the Internet were also asked.

All respondents were interviewed by telephone 1 month after they had their mammogram. They were invited to join a follow-up study and were asked to select the version of questionnaire they preferred.

The sample size was calculated to provide a statistical power of at least 90% to detect a true difference in response rate of 15%. The actual power was 93.8%. Women had an equal probability of assignment to the two groups. The randomization code was developed using a computer random number generator. We tested the significance of categorized variables by the chi-square test and compared continuous variables by risk differences with 95% confidence intervals. Homogeneity across strata was tested with the Mantel-Haenszel test.

## Results

The characteristics of the invited women are shown in Table 1. Approximately 80% of the women were between 30 and 59 years old. The distributions within the two randomized groups were similar with respect to age, place of residence, and category of referral.

**Table 1:** Characteristics of patients, by randomization group

	Internet Group, % (n = 257)	Paper-and-Pencil Group, % (n = 276)
**Age (years)**		
20-29	5.1	7.4
30-39	25.0	21.0
40-49	29.7	29.6
50-59	26.8	27.6
60-67	13.4	14.4
		
Place of Residence^*^		
Rural	60.1	53.3
Village/suburb	26.5	27.2
Urban	13.4	19.5
		
**Category of Referral^†^**		
Acute	50.0	47.9
Subacute	18.5	20.6
Nonacute	31.5	31.5

^*^Defined by postal code

^†^The acute group was called in for mammography within 3-14 days, the subacute group within 1 to 3 weeks, and the nonacute group, not before 5 months.

The response rate before the reminder was 17.9% in the Internet group compared to 73.2% in the paper-and-pencil group, corresponding to a 55.3% difference in response rate in favor of the paper-and-pencil version (Table 2). The same tendency was found in all strata with respect to age, place of residence, and category of referral (see Table 2).

After the reminder, the response rate improved distinctly in the group originally randomized to the Internet (Table 3). Among the women assigned to the nonacute group, who had the longest respite before their mammogram, the response rate was even higher in the group randomized to the Internet version.

The completeness of answers in the two versions is summarized in Table 4. The Internet version produced significantly more complete questionnaires than the paper-and-pencil version. For the paper-and-pencil version, there was a tendency toward more incomplete scales the longer the scales were.

**Table 2:** Response rate before reminder, by randomization group

	Internet Group, % (n = 257)	Paper-and-Pencil Group, % (n = 276)	Difference, % (95% CI)^*^
**Total**	17.9	73.2	55.3 (48.3-62.3)
**Age (years)**			
20-29	10.5	85.7	75.2 (52.2-98.1)
30-39	16.7	72.5	55.8 (41.3-70.3)
40-49	23.7	68.3	44.6 (30.7-58.5)
50-59	18.3	75.7	57.4 (44.1-70.7)
60-67	10.8	75.7	64.9 (47.8-81.9)
			*χ*^2^_4_ = 4.8, *P* = .30
**Place of Residence^†^**			
Rural	17.5	74.7	57.2 (48.0-66.49)
Village/suburb	22.9	71.2	48.4 (34.1-62.7)
Urban	12.0	70.3	58.3 (41.0-75.5)
			*χ*^2^_2_ = 2.2, *P* = .33
**Category of Referral^‡^**			
Acute	23.9	72.5	48.9 (38.3-59.5)
Subacute	13.2	70.6	57.4 (41.9-72.9)
Nonacute	12.4	75.9	63.5 (52.0-75.0)
			*χ*^2^_2_= 5.1, *P* = .08

^*^With Mantel-Haenszel test of homogeneity

^†^Defined by postal code

^‡^The acute group was called in for mammography within 3-14 days, the subacute group within 1 to 3 weeks, and the nonacute group, not before 5 months.

**Table 3:** Response rate after reminder, by randomization group

	Internet Group (n = 257)		Paper-and-Pencil Group (n = 276)		Difference in % (95% CI)
	%	% Reminded^*^ (No.)	%	% Reminded (No.)	
**Total**	64.2	75 (159)	76.5	59 (44)	12.2 (4.5-20.0)
**Category of Referral**^†^					
Acute	49.6	58 (55)	74.6	39 (15)	25.0 (13.6-36.5)
Subacute	67.9	76 (35)	76.5	53 (8)	8.5 (−8.6-25.7)
Nonacute	84.0	97 (69)	79.3	100 (21)	−4.6 (−16.3-7.0)

^*^The percentage of primary nonrespondents who were reminded

^†^The acute group was called in within 3-14 days, the subacute group within 1 to 3 weeks, and the nonacute group, not before 5 months.

**Table 4:** Completeness of the three scales, by version

	Internet Version, % (n = 46)	Paper-and-Pencil Version, % (n = 202)	Difference, % (95% CI)
Total	97.8	63.4	34.5 (26.6-42.3)
Short Form-36 [16] (36 items)	100.0	71.3	28.7 (22.5-35.0)
Multidimensional Fatigue Inventory-20 [17] (20 items)	97.8	90.6	7.2 (1.4-13.1)
Hospital Anxiety and Depression Scale [18] (14 items)	100.0	92.6	7.4 (3.8-11.0)

The scores for the eight subscales of Short Form-36 are displayed in Table 5. There were no statistically significant differences between the two versions.

**Table 5:** Scores for subscales of Short Form-36, by randomization group

Subscale of Short Form-36 [16]	Internet Version, Mean (SD)	Paper-and-Pencil Version, Mean (SD)	Difference		
			%	*t*	*P*

Physical Function	91.4 (15.1)	90.1 (15.6)	1.3	0.5	.60
Role Physical	85.9 (30.6)	81.9 (30.3)	3.9	0.8	.43
Bodily Pain	81.2 (22.2)	76.3 (21.1)	4.9	1.4	.16
General Health	81.3 (14.7)	77.1 (18.7)	4.2	1.4	.16
Vitality	65.4 (22.7)	64.1 (22.2)	1.3	0.4	.71
Social Function	89.9 (16.4)	87.3 (19.0)	2.6	0.9	.39
Role Emotional	86.2 (25.9)	78.7 (32.8)	7.5	1.4	.14
Mental Health	75.2 (17.3)	71.8 (19.8)	3.4	1.1	.28

During the telephone interview with the respondents 1 month after they had their mammogram, they were invited to participate in the follow-up part of the study. They were asked to select the version of future questionnaires they preferred. The majority (55.4%) preferred the paper-and-pencil version, while 32.4% preferred the Internet version. The remaining 17.1% declined further participation. Among the 46 respondents from the Internet group, 73.2% preferred to continue on the Internet compared to 17.1% who preferred to change to a paper-and-pencil version.

Access to Internet, estimated by answers from the paper-and-pencil group, is displayed in Table 6.

**Table 6:** Internet access among the paper-and-pencil group

	No.	At Home, %	Other^*^, %	None, %
**Total**	198	68.7	9.6	21.7
**Age (years)**				
20-29	12	58.3	25.0	16.7
30-39	48	70.8	8.4	20.8
40-49	56	82.1	3.6	14.3
50-59	55	63.6	18.2	18.2
60-65	27	51.9	0.0	48.1
*χ*^2^_8_= 25.6, *P* = .001				
**Place of Residence^†^**				
Rural	120	62.5	12.5	25.0
Village	52	86.5	7.7	5.8
Urban	26	61.5	0	38.5
*χ*^2^_4_= 17.1, *P* = .002				
**Education Level (years)^‡^**				
7-10	33	39.4	6.1	54.5
10-12	47	68.1	4.3	27.6
13-17	113	77.0	13.3	9.7
*χ*^2^_4_= 33.2, *P* < .001				

^*^At work, local library, etc

^†^Defined by postal code

^‡^According to International Standard Classification of Education

## Discussion

We found an initial response rate of only 17.9% in the Internet group compared to 73.2% in the paper-and-pencil group. However, after a reminder, when the participants were free to choose between versions, the total response rate was similar in the two randomized groups. The quality of data regarding completeness was superior in the Internet version for all the involved scales. We did not identify any differences in Short Form-36 subscales. However, even in a randomized study, caution should be exercised when comparing the distribution of answers between the two groups since the distributions depend on differences in the two methods as well as selection bias, especially when the response rate in one of the groups is very low.

The population was unselected with respect to Internet access and experience. According to the 2005 Statistics Denmark survey, 77% of Danish women had access to the Internet [19]. Based on answers from the paper-and-pencil group, we estimate that 70% of the women in the present study had access to the Internet at home. Access was closely associated with level of education. The geographic area surrounding the public hospital includes rural locations as well as the fifth largest city in Denmark. We consider our sample representative for female patients in Denmark.

The most prominent weakness of the Internet version was a low response rate, and we could not identify any single determinant factor. However, as expected, the response rate was highest in the age group with greatest access to the Internet. After a reminder letter, which stated that participants were free to fill out their preferred version of the questionnaire, the total response rates were nearly the same. However, women in the acute and subacute groups had less time to complete the questionnaire before their mammogram, which in some cases prevented the reminder.

Response rates to Internet questionnaires reported in the literature vary a lot between studies [1,6-14]. It is evident that studies conducted in populations with known access to the Internet are supposed to have higher response rate than studies of populations without known access, like the present study. However, differences in response rate may also be attributed to methodology and other characteristics of the population. A Swedish study compared the same paper-and-pencil questionnaire in two different versions with respect to ordering of questions and level of difficulty and found that the proportion of completers varied significantly [20]. It is plausible that populations of patients and general population samples may react differently to an invitation to complete a Web questionnaire about health-related issues.

The fact that only 17.1% of respondents in the Internet group preferred to shift to the paper-and-pencil version when asked to join the follow-up study indicates that Internet versions may be more feasible in follow-up studies. One advantage of the Internet version is a high degree of completeness, and the design of Internet questionnaires allows the researcher to compensate for human error among participants who enter inconsistent answers or accidentally skip an item or even a page.

At present, Internet questionnaires can hardly stand alone as the method of data collection in studies of patients. Access to the Internet still depends on socioeconomic factors, and results obtained solely from Internet users may be biased. The general population must become more familiar with the Internet before an online survey can be the first choice of researchers, although it is worthwhile considering within selected populations of patients as it saves resources and provides more complete answers. An Internet version may be combined with a traditional version, and it may be more feasible to offer Internet versions in follow-up studies.
